# Uterine Sarcomas: Are There MRI Signs Predictive of Histopathological Diagnosis? A 50-Patient Case Series with Pathological Correlation

**DOI:** 10.1155/2021/8880080

**Published:** 2021-07-01

**Authors:** Siegfried Hélage, Stéphanie Vandeventer, Jean-Noël Buy, Corinne Bordonné, Pierre-Alexandre Just, Denis Jacob, Michel Ghossain, Pascal Rousset, Élisabeth Dion

**Affiliations:** ^1^Department of Radiology, Hôtel-Dieu de Paris (AP-HP), 1 Place Du Parvis Notre-Dame, Paris 75004, France; ^2^Department of Pathology, Hôpital Cochin (AP-HP), 27 Rue Du Faubourg Saint-Jacques, Paris 75014, France; ^3^Department of Gynecological Surgery, Clinique Bizet, 21 Rue Georges Bizet, Paris 75016, France; ^4^Department of Radiology, CHU Hôtel-Dieu de France, Beirut, Lebanon; ^5^Department of Radiology, Centre Hospitalier Lyon Sud, Hospices Civils de Lyon, Pierre Bénite, France

## Abstract

**Purpose:**

To make clear distinction between two radiological types of uterine sarcomas.

**Methods:**

50 preoperative MRI were analyzed retrospectively, blinded to histopathology: 11 endometrial stromal sarcomas (ESS), 19 leiomyosarcomas (LMS), 18 carcinosarcomas/malignant mixed Mullerian tumors (MMMT), and 2 smooth muscle tumors of uncertain malignant potential (STUMP).

**Results:**

According to their locations, two radiological types of sarcomas were identified: type 1: intracavitary (ESS, MMMT) and type 2: intramyometrial (LMS, STUMP). In both types, all tumors displayed intermediate T2-weighted signal (*p* < 0.001) and high diffusion-weighted imaging (DWI) b1000 signal (*p* < 0.001). Dynamic contrast-enhanced (DCE) MRI showed intratumoral pathologic vessels (98%) and heterogeneity at venous phase (*p* < 0.001). In the type 1 subgroup, all tumors displayed local spread: invasion of junctional zone on T2-weighted imaging (T2WI), irregular margins on DWI, and disruption of arcuate arteries subendometrial ring on DCE-MRI. In the type 2 subgroup, all tumors displayed irregular margins on T2WI, DWI, and DCE-MRI. Tumor heterogeneity was due to necrosis (*p* < 0.001). Most commonly the tumor was single (61%). In both types, apparent diffusion coefficient (ADC) lesser than or equal to 0.86 × 10^−3^ mm^2^/s (sensitivity = 73%, specificity = 92%) was suggestive of malignancy.

**Conclusion:**

It may be feasible to get close to histological type of a uterine sarcoma based on our topographic classification into two radiological subgroups, corresponding to two kinds of diagnostic difficulties. *Advances in knowledge*. MRI signs suggestive of histopathological malignancy are identifiable, considering the triad T2WI/DWI/DCE-MRI, easily for type 1 but less easily for type 2; the threshold value for ADC is 0.86 × 10^−3^ mm^2^/s.

## 1. Introduction

Uterine sarcomas are very rare neoplasms with poor prognosis. They account for only 1 to 3% of gynecological tumors and 3 to 7% of uterine tumors. The 5-year survival rate in advanced stages is less than 10% [1, 2]. In the literature, radiological series include very few patients. Besides, authors never make a distinction between endometrial sarcomas and myometrial sarcomas [3–5]. MR features predictive of histological malignancy have not been much evaluated, especially a combination of several signs.

Considering myometrial tumors, pathological distinction between benign and malignant types is often difficult to such an extent that there are borderline types termed “STUMP” for which only tumor progression during follow-up will allow physicians to conclude [6].

The aim of the present observational study was to define two types of uterine sarcomas based on tumor macroscopic location within the uterus, found to be closely related to pathological subtypes of uterine sarcomas. Thus, we assessed MR features predictive of histological malignancy for each of these two location types. Lastly, we pointed out the value of DCE-MRI, especially concerning angiograms analysis, to determine the malignant character of the tumor and histological differential diagnoses.

## 2. Materials and Methods

### 2.1. Study Population

After institutional ethical board approval, our study population included retrospectively 50 women, aged 20–95 years (mean age 60) who underwent MRI for various uterine masses before surgery, in 6 medical centers in France, from October 2009 to July 2016.

All patients underwent surgery (colpohysterectomy with or without bilateral adnexectomy). The diagnosis of all tumors was proved at pathology.

Pathological diagnosis for all these uterine sarcomas consisted of ESS (*n* = 11), MMMT (*n* = 18), LMS (*n* = 19), and STUMP (*n* = 2).

By convention, in patients with multiple leiomyomas, the uterine tumor was considered to be single if there were ≤3 leiomyomas ≤20 mm in diameter.

### 2.2. MRI Protocol

MRI were performed on 1.5 T systems (Achieva®, Philips Healthcare, Netherlands; Aera®, Siemens Healthcare, Germany; Signa®, GE Healthcare, USA) using a phased array pelvic coil. Protocols included sagittal and axial spin echo T2WI and axial spin echo T1WI with or/and without Fat-Sat.

Diffusion-weighted imaging (DWI) was acquired in 37 patients in the axial plane using single-shot EPI. *b* values were 0 and 1000 s/mm^2^. ADC maps were calculated in these 37 patients.

DCE-MRI was performed for 9 of 11 ESS, 16 of 18 MMMT, 15 of 19 LMS, and for all STUMP. DCE-MRI used axial 3D GRE sequence. 20 acquisitions of 72 slices each requiring 23 seconds per acquisition were acquired after intravenous injection of 0.1 mmol/kg body weight of gadoterate meglumine (Dotarem®, Guerbet, France).

### 2.3. Image Analysis

MRI were retrospectively analyzed by three independent radiologists (30, 7, and 4 years of experience respectively), blinded to the final histological diagnosis.

MRI were reviewed regarding the following items: size of the tumor (greatest diameter), isolated character of the tumor (i.e., single character), topography of the uterine lesion (myometrium or endometrium), tumor margins (blurred or sharp, irregular or regular), presence of intratumoral necrosis or hemorrhage, T2 and DWI signal intensity (with ADC mapping) of the solid component, the heterogeneous character of the tumor, vascular enhancement profile and angiograms (tumoral vessels), and intrauterine and extrauterine extension (adenopathy, peritoneal effusion, peritoneal nodules of carcinomatosis).

An intermediate T2 signal was defined as a signal between pelvic muscle and adipose tissue, and a T2 signal was considered high if similar to that of bladder urine.

A high b1000 signal intensity was defined as a higher signal than the myometrium. For diffusion analysis, we recorded the ADC values of each tumor by placing a circular region of interest (ROI) on the ADC map in the area with the lowest ADC, avoiding hemorrhagic, necrotic, or cystic portions within the lesion by referring to T1-weighted, T2-weighted, and contrast-enhanced images. Several ROI were placed on the maps, and the lowest value of mean ADC was recorded.

DCE-MRI evaluated the presence of tumoral vessels at arterial phase and percentage of necrosis at venous phase and later phases.

We defined two radiological types of uterine sarcomas:

- Type 1: endometrial epicenter or protruding into the endometrial cavity
- Type 2: myometrial epicenter

### 2.4. Pathological Correlation

Diagnosis of all malignant lesions was established at pathological examination in each center, with macroscopy, microscopy, and immunohistochemistry.

### 2.5. Statistical Analysis

With regard to the number of patients, the following nonparametric tests were used to determine *p* values: Fisher's exact test for qualitative variables and Mann–Whitney test for quantitative variables. The reference benign mesenchymal tumor population (leiomyomas) considered was that of Lin's study [4], taken as a historical control population used for comparison. The receiver operating characteristics (ROC) curve and Youden index, integrating both sensitivity and specificity, were used to identify the best threshold for ADC.

A *p* value < 0.05 was considered statistically significant. XLSTAT software was used for all statistical analysis.

## 3. Results

### 3.1. Correlation between Location within the Uterus and Histological Type

In the type 1 subgroup, among the 27 tumors, there were 9 ESS (33%), 18 MMMT (67%), but neither LMS nor STUMP. In the type 2 subgroup, among the 23 tumors, there were 19 LMS (83%), 2 STUMP (9%), 2 ESS (9%), and no MMMT. This topographic feature was statistically significant (*p* < 0.001) (Table 1).

### 3.2. Lesion Features

Greater diameters of tumor burdens on MRI ranged from 10 mm to 240 mm (mean 86 mm, median 70 mm).

**Figure 1:**
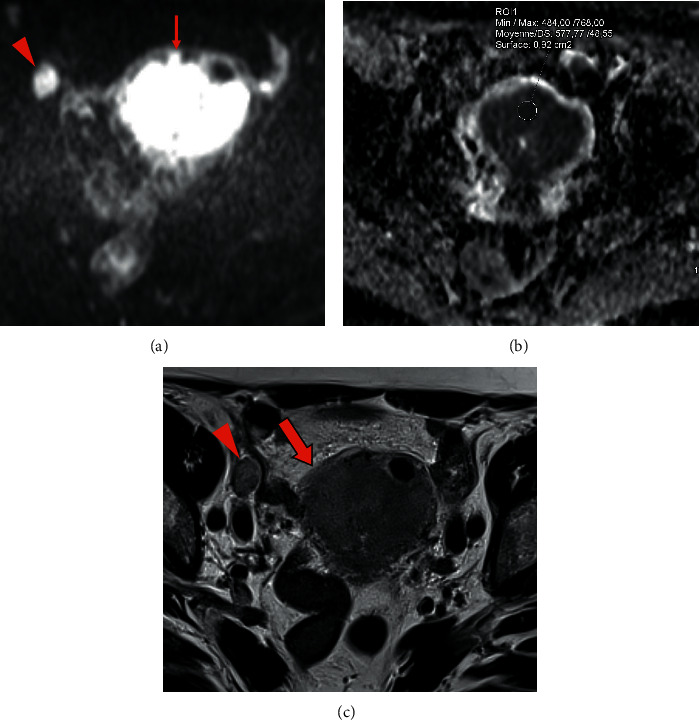
Pelvic MRI of endometrial stromal sarcoma: (a, b, c) axial DWI b1000 showing a large intrauterine tumor with high signal intensity and lobular/irregular tumor contours (arrow) (a) and low ADC value (0.58 × 10^−^³ mm^2^/s) (b), and an obturator lymph node metastasis (arrowhead) (a). Note the intermediate T2 signal intensity of these two lesions and the blurred contours of the tumor (empty arrow) (c).

**Figure 2:**
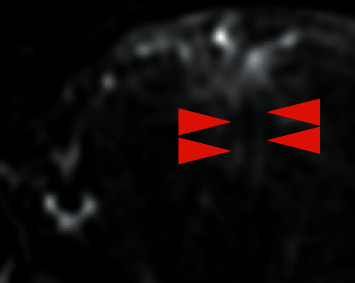
Pelvic MRI of endometrial stromal sarcoma: in DCE-MRI, angiogram depicted tumor regions containing dense wide, regular, and parallel vessels (arrowheads).

In both types, all tumors displayed an intermediate or high T2 signal (50/50, 100%, *p* < 0.001) and a high b1000 signal (37/37, 100%, *p* < 0.001) (Table 2). Sometimes, DWI allowed easy detection of metastatic regional lymph nodes (Figure 1). Angiographic features assessment showed an increase in the number of arteries around the tumor and the presence of pathologic vessels within it. In most cases (41/42, 98%), the vascularity was greater in the tumor than in the surrounding tissues, some tumor regions containing very dense wide, regular, and often parallel vessels (“combed” vessels) (Figure 2). In one case, vessels were not visible, coinciding with a low-grade ESS. A majority of tumors (44/49, 90%, *p* < 0.001) showed heterogeneity at venous phase (Table 2).

**Figure 3:**
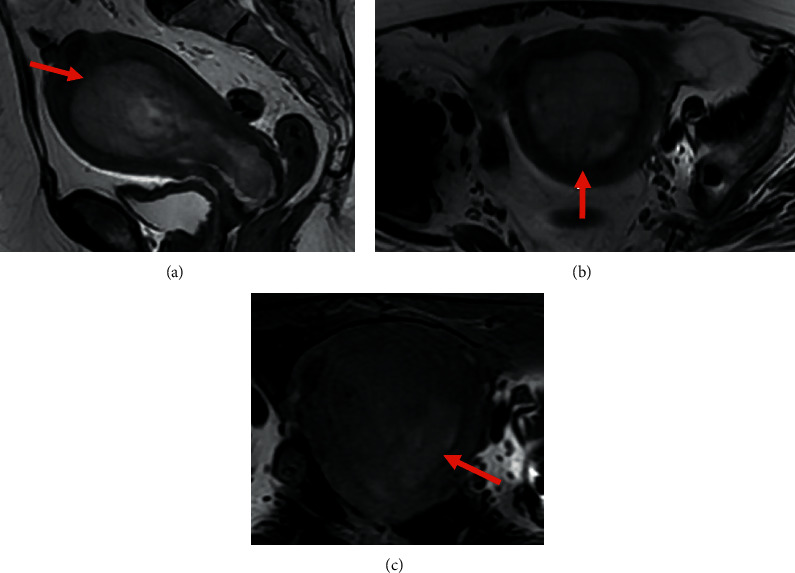
Pelvic MRI of endometrial stromal sarcoma: (a, b, c) sagittal (a) and axial (b) T2WI showing a large intrauterine tumor with heterogeneous intermediate T2 signal with invasion of junctional zone (arrow); and axial T1 Fat-Sat WI (c) showing high signal intensity intratumoral hemorrhage (arrow).

**Figure 4:**
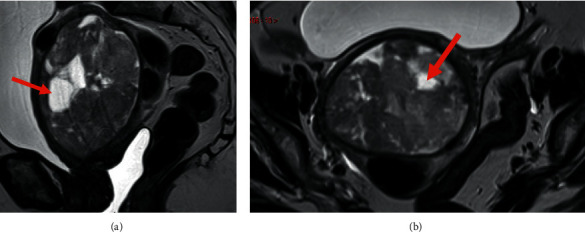
Pelvic MRI of MMMT: (a, b) sagittal (a) and axial (b) T2WI showing a large intrauterine tumor with mixed heterogeneous signal, cystic component showing high signal intensity (arrow).

**Figure 5:**
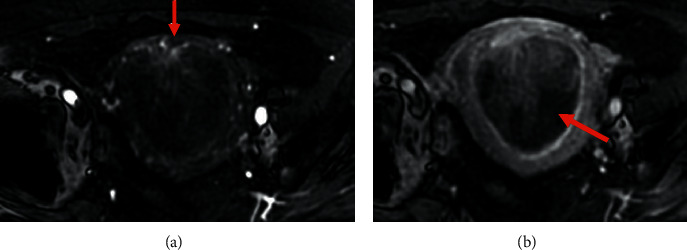
Pelvic MRI of endometrial stromal sarcoma: (a, b) axial DCE-MRI showing tumoral vessels in the lesion at arterial phase and disruption of arcuate arteries subendometrial ring (arrow) (a) and percentage of necrosis at venous phase (arrow) (b).

**Figure 6:**
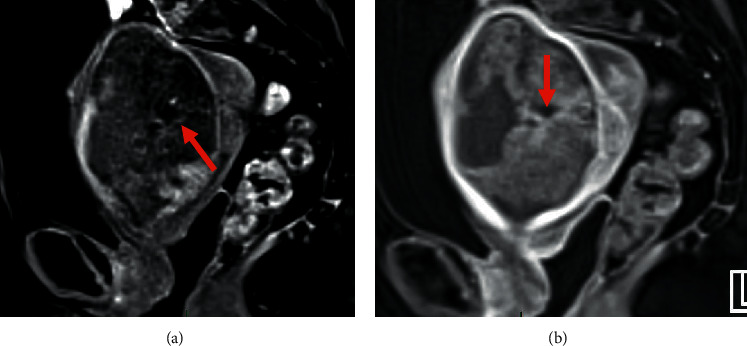
Pelvic MRI of MMMT: (a, b) sagittal DCE-MRI evaluated the presence of tumoral vessels in the lesion at arterial phase (arrow) (a) and percentage of necrosis at venous phase (arrow) (b).

In the type 1 subgroup, all tumors (27/27, 100%) displayed signs of local spread: invasion of junctional zone on T2WI (Figures 1, 3, and 4), irregular margins on DWI (Figure 1), and disruption of the arcuate arteries subendometrial ring (Figures 5 and 6).

**Figure 7:**
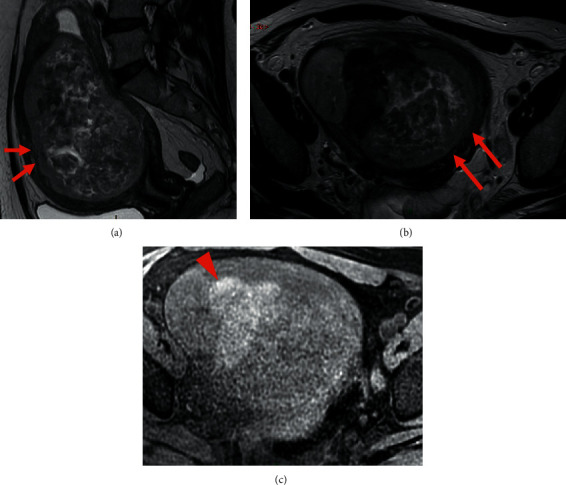
Pelvic MRI of leiomyosarcoma: (a, b, c) sagittal (a) and axial (b) T2 and axial (c) T1 Fat-Sat WI showing a large uterine tumor developed within the myometrium, showing heterogeneous intermediate T2 signal with irregular margins (arrows) and intratumoral hemorrhage in high T1 Fat-Sat WI signal (arrowhead) (c).

**Figure 8:**
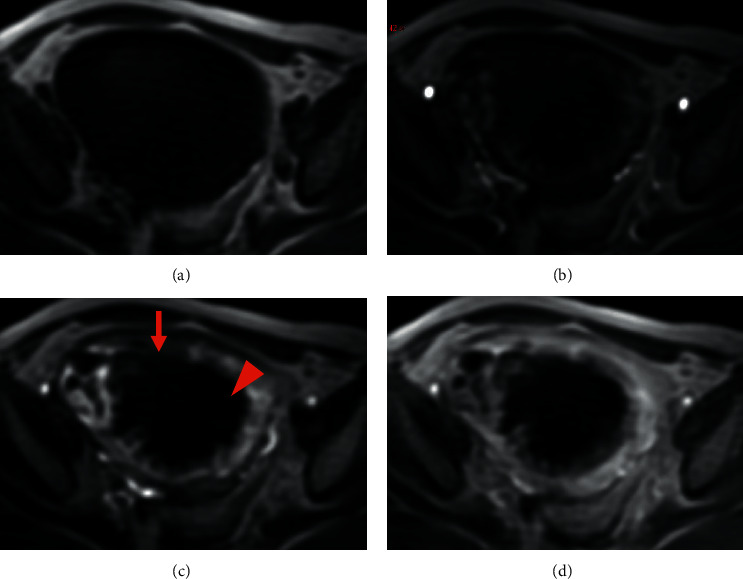
Pelvic MRI of leiomyosarcoma: (a, b, c, d) axial DCE-MRI without injection (a), at arterial phase (b), venous phase (c), and late phase (d). Note the disruption of the peripheral vascular ring (arrow) and heterogeneity due to central necrosis (arrowhead) (c).

In the type 2 subgroup (Tables 2 and 3), considering LMS and STUMP compared with leiomyomas, all tumors displayed an intermediate or high signal on T2WI (21/21, 100%, *p*=0.005). A majority of tumors (14/21, 67%) displayed irregular margins on T2WI, on DWI, and on DCE-MRI (disruption of the peripheral vascular ring). Tumor heterogeneity was due to intratumoral hemorrhage (8/20, 40%, *p*=0.755) visible as a high signal on T1WI with Fat-Sat, necrosis (17/20, 85%, *p* < 0.001) visible as areas of nonenhancement at venous phase, and to a lesser extent, to myxoid changes (2/21, 9%) visible as large areas with a high T2 signal (Figures 7 and 8). An isolated tumor (i.e., single character) was common (13/21, 61%).

### 3.3. ADC Maps

**Figure 9:**
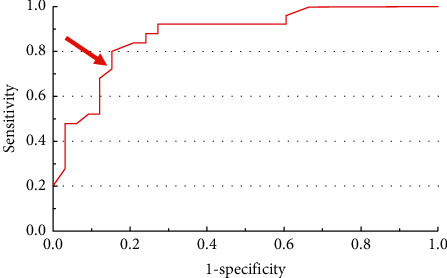
ROC (receiver operating characteristic) curve of ADC value for the risk of being malignant. Youden index reaches its maximum value (YI = 0.655) for a threshold lesser than or equal to 0.86 × 10^−^³ mm^2^/s (arrow) (sensitivity = 73%; specificity = 92%).

ADC values ranged from 0.41 × 10^−^³ mm^2^/s to 1.3 × 10^−^³ mm^2^/s (mean 0.79 × 10^−^³ mm^2^/s, median 0.77 × 10^−^³ mm^2^/s), with a maximum Youden index (YI = 0.655) suggestive of malignancy for a threshold lesser than or equal to 0.86 × 10^−^³ mm^2^/s (Figure 9) (sensitivity = 73%, specificity = 92%, PPV = 92%, OA = 81%). Overall accuracy reaches its maximum value (OA = 83%) for a threshold of 1.05 × 10^−^³ mm^2^/s (sensitivity = 85%; specificity = 80%, YI = 0.653).

## 4. Discussion

We defined two radiological types of uterine sarcomas on MRI, based on their location within the uterus, matching with two different histological categories of sarcomas. Type 1 sarcomas are of endometrial epicenter; they are protruding into uterine cavity and have the potential to invade the subendometrial myometrium macroscopically. In our study, these type 1 sarcomas matched almost exclusively with ESS and MMMT on pathological examination. Type 2 sarcomas are of myometrial epicenter and stay confined to the myometrium. In our study, these type 2 sarcomas matched exclusively with LMS and STUMP on pathological examination. Defining these two radiological types of sarcomas, corresponding clearly to two different histological categories of sarcomas, will have a number of implications during the diagnostic process and prognostic assessment. The malignant character of type 1 tumors is easy to diagnose, considering the T2WI/DWI/DCE-MRI triad [3–5, 7]. On these three sequences, disruption of the endometrial-myometrial interface is usually easy to demonstrate, in addition to the criteria of malignancy related to each sequence we found statistically significant in our study. These criteria were an intermediate signal on T2WI, a high DWI b1000 signal increasing compared with b0, and visible intratumoral vessels of malignant type on DCE-MRI. The greater ease in determining the proper diagnosis at radiological step for type 1 tumors is also found at pathological step. In contrast, the malignant character of type 2 tumors is more difficult as analysis of their contours is more subjective on those three MRI sequences. For type 2 tumors, diagnostic issues are also seen in the pathological process owing to, among other things, the lack of stromal reaction; pathologists circumvent this pitfall, talking about “smooth muscle tumors of uncertain malignant potential” (STUMP) [6]. Frequency of intratumoral T1 Fat-Sat WI hypersignal related to hemorrhage was not significantly different between malignant and benign mesenchymal tumors. We believe this is because coagulation tumor cell necrosis visible in sarcomas at pathological examination is by its nature distinct from hyaline necrosis of ischemic type visible in benign mesenchymal tumors [6].

At the pathological level, potential diagnostic errors are possible in determining the type of sarcoma. Usually, pathological diagnostic is dependent on a good macroscopic examination of the resected gross specimen; this analysis makes it possible to identify the most informative areas to sample for microscopic examination. Extensive use of immunohistochemistry could lead to rush this crucial step of macroscopic examination, causing sampling biases. But, according to Hart [2], an endometrial stromal sarcoma can contain up to 30% of smooth muscle tissue without however being included in leiomyosarcomas. Also, according to Blaustein [6], the portion of the epithelial component shall be at least 10% to have an MMMT diagnosed; a poor resected gross specimen sampling during macroscopic examination could lead to miss this component.

In our study, for the two radiological types of sarcomas, intermediate signal intensity (i.e., gray) on T2WI is highly suggestive of malignancy. However, intratumoral hemorrhage on T1WI with Fat-Sat is not a significant predictor of malignancy, especially for LMS and STUMP.

For type 2 sarcomas, intermediate signal intensity on T2WI is sensitive but not specific for malignancy. Indeed, degenerated leiomyomas, especially edematous ones, may display a high T2WI signal. At the pathological level, on macroscopic examination, broadly speaking, a leiomyoma is white and stiff, whereas a leiomyosarcoma is gray and smooth. So it makes sense that these macroscopic features would lead to different signals on T2WI. In our study, there were no sarcomas with a low signal on T2WI equal to that of pelvic muscles. In other words, a low signal intensity on T2WI has a good negative predictive value for malignancy. Furthermore, in our study, most type 2 sarcomas were found within the uterine wall as single tumors. In a polymyomatous uterus, a small leiomyosarcoma lost in the major benign component could be missed; this malignant focus may be the source of a future tumor recurrence.

In our study, DWI sequence presents several advantages. Features in favor of a malignant nature are a high signal intensity at high *b* values, increasing from b0- to b1000-weighted sequence, and lobular or irregular tumor contours. Furthermore, DWI allows detection of regional lymph nodes or peritoneal metastases that could be missed on conventional T2WI sequence. This property is analogous to a “scintigraphic effect”.

In our study, an ADC value lower or equal to 0.86 × 10^−^³ mm^2^/s is highly suggestive of malignancy (sensitivity = 73%, specificity = 92%, PPV = 92%). Our threshold is lower than that found in Namimoto's study (1.05 × 10^−^³ mm^2^/s), Lin's study (1.08 × 10^−^³ mm^2^/s), and Thomassin's study (1.23 × 10^−^³ mm^2^/s) [3–5]. As an illustration of the benefit of measuring ADC values, the only type 2 tumor that contours were regular and sharp on T2WI showed a high signal on DWI b1000 sequence, with an ADC value of 0.84 × 10^−^³ mm^2^/s; it was a low-grade LMS in pathology.

Uterine myxoid leiomyosarcoma is an infrequent but distinct malignant neoplasm. By convention in pathology, a tumor is designated as myxoid if the neoplasm contains an “abundant” myxoid extracellular matrix occupying at least 50% of the tumor area. Morphologic features typical of conventional LMS tend to be subtle in the myxoid counterpart particularly when the myxoid matrix is extensive [6]. In our experience, a myxoid leiomyosarcoma may show a lack of restricted diffusion. However, an infiltrative tumor border is typically present and should be assessed routinely as a diagnostic criterion for malignancy.

Angiography on DCE-MRI displays a lower quality than that of conventional angiography in terms of imaging contrast and spatial resolution. However, in our study, for the two radiological types of sarcoma, DCE-MRI allowed detection of intratumoral vessels at arterial phase and disruption of the vascular circle surrounding the tumor, features suggestive of malignancy, along with contrast washout. For type 1 sarcomas, this vascular circle corresponds to subendometrial arcuate arteries network; for type 2 sarcomas, it corresponds to peritumoral vascular ring. At venous phase and on delayed sequences, heterogeneity due to intratumoral necrosis could be assessed, a feature typically found in malignant tumors, due to mismatch between cell proliferation and angiogenesis.

Interestingly, as previously reported in the literature [8–11], in our study, sarcomas' angiograms were different from those of carcinomas. Angiograms depicted tumor regions containing very dense wide, regular, and often parallel vessels (“combed” vessels) in sarcomas. For carcinomas, angiograms usually depict a network of fine multiple and tortuous intratumoral vessels.

**Figure 10:**
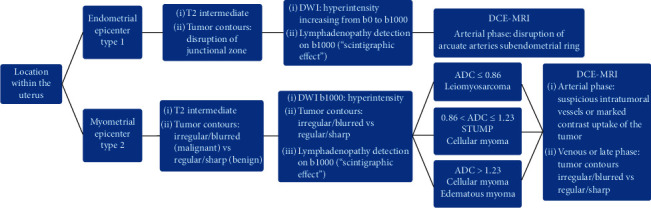
Diagnostic algorithm to assess malignant MR features and to suggest pathological hypotheses. For type 2 tumors, the radiologist needs ADC values only if the lesion has a high b1000-weighted signal.

To our knowledge, our series of uterine sarcomas is the largest in the literature and includes a similar number of each histological subtype, namely ESS, MMMT, and LMS. Other series included few LMS, and a majority of ESS in Thomassin's study [5]. For the first time, our study distinguishes two radiological types of uterine sarcomas, which match with two different macroscopic aspects in pathology and two distinct histological categories. Furthermore, difficulties in diagnosis are different between these two radiological types. Thus, MRI reading should begin with locating tumor epicenter within the uterus (protruding into the endometrial cavity versus myometrium) to classify in type 1 or 2. Then, MRI features of malignancy should be sought, the triad T2WI/DWI/DCE-MRI being the most efficient way; hence, we here propose a diagnostic algorithm (Figure 10).

Diagnosis of malignancy is easy to assert on MRI for type 1 tumors. In contrast, radiologists' conclusions should be circumspect for type 2 tumors, for which difficulties remain in distinguishing between benign and malignant processes (i.e., leiomyoma versus leiomyosarcoma). For type 2, in particular, several informative MRI sequences are required to have many arguments, thereby reducing the risk of diagnostic errors and allowing suitable treatment to be performed. Considering this issue for type 2 tumors, in order to select patients with very likely malignant subtypes preoperatively, especially to distinguish atypical leiomyomas from leiomyosarcomas, it may be feasible to design an MRI scoring system using the features found to be significant in our univariate analysis, as has already been done for other mesenchymal tumors [12, 13].

Our study has a few limitations. Our study population is the largest in the literature for such rare tumors; however, multivariate analysis was impractical. The retrospective aspect of this multicenter study explains variable scanning techniques, sometimes without DWI and/or DCE-MRI. Pathology procedures might vary between institutions. The criterion we used for malignancy assessment was initial histopathology report conclusions, notwithstanding follow-up data.

In the uterine smooth muscle tumors group, myomas proportion is higher than 99.5%, and leiomyosarcomas frequency is less than 0.5%. Given this epidemiological imbalance, constitution of a matched comparison group of 21 myomas for our 21 LMS/STUMP would have yield a sample selection bias due to the limited number of tumors. In order to avoid this bias, we decided to consider a series of myomas, which have been previously validated in the literature, namely Lin's series [4]. MRI features of these myomas were accurately described by Lin aiming at comparison with LMS/STUMP. The number of myomas in this reference series is 25, almost identical to the number of LMS/STUMP in our study. This similarity allowed an optimum statistical matching. The principle of using a reference series validated in the literature is acceptable since it is admitted in meta-analyses and in epidemiological studies, such as the use of the Framingham cohort in vascular medicine for example.

## 5. Conclusion

We have identified several MRI features specific to the different histological subtypes of uterine sarcomas, whose base is firstly tumor macroscopic location within the uterus: endometrial or myometrial epicenter.

In addition to intellectual interest for radiologists with the view to propose a pathological diagnostic hypothesis, this MRI reading approach would allow suitable patient management to be performed.

An endometrial neoplasm (type 1 tumor), which features of malignancy are easy to detect, will be treated by wide surgical excision; the first hypothesis would be carcinoma.

In contrast, for myometrial neoplasms (type 2 tumors), to discriminate between leiomyoma and sarcoma is not that simple, even in pathology, some pathologists talking about “tumors of uncertain malignant potential” (STUMP). Hence, for type 2 tumors, a combination of several MRI signs suggestive of malignancy, if present, will all the more compel physicians to propose surgical treatment instead of follow-up. Moreover, in patients with comorbidities that increase anesthetic risk, presence of such MRI signs will provide a valid argument supporting surgery during tumor boards, especially now that prosecution of medical professionals is becoming increasingly common. Concerning histological diagnosis, such MRI features suggestive of malignancy will strongly incite the pathologist to fulfill the widest possible sampling of the resected gross specimen in order to find the small malignant focus lost in the major benign and/or fibrous component. This focus will be the source of a future tumor recurrence. This is for producing the most accurate diagnosis possible. Even if pathology report is reassuring, such MRI features will provide a strong argument to plan a long-term follow-up to check for evidence of relapse.

## Figures and Tables

**Table 1:** Tumor macroscopic location within the uterus on MRI.

	SSE, MMMT	LMS, STUMP
Number of tumors, % (*n*)	58 (29)	42 (21)
Type 1, % (*n*)	93 (27/29)	0 (0/21)
Type 2, % (*n*)	7 (2/29)	100 (21/21)

**Table 2:** Evaluated MRI features for both type 1 and type 2 uterine sarcomas.

Intermediate or high T2 signal, % (*n*)	100 (50/50)
High b1000 signal on DWI, % (*n*)	100 (37/37)
Blurred/irregular margins or invasion of junctional zone on T2WI, % (*n*)	80 (40/50)
Adenopathy on DWI, % (*n*)	39 (14/36)
Visible intratumoral vessels or contrast uptake at arterial phase, % (n)	98 (41/42)
Tumor heterogeneity at venous phase, % (*n*)	90 (44/49)
Blurred/irregular margins at venous phase, % (*n*)	78 (38/49)

**Table 3:** Univariate analysis of MRI-specific features for type 2 uterine sarcomas.

	LMS, STUMP	*p*
Intermediate or high T2 signal, % (*n*)	100 (21/21)	0.005^*∗*^
High b1000 signal on DWI, % (*n*)	100 (15/15)	0.015^*∗*^
High signal area on T1WI with fat suppression, % (*n*)	40 (8/20)	0.755
Tumor heterogeneity at venous phase, % (*n*)	85 (17/20)	<0.001^*∗*^

^*∗*^Indicates statistically significant different values between leiomyomas and leiomyosarcomas or tumors of uncertain malignant potential with *p* values less than 0.05.

## Data Availability

The data used to support the findings of this study are available from the corresponding author upon request in order to protect patient privacy.

## Abbreviations

ADC: Apparent diffusion coefficient
DCE: Dynamic contrast-enhanced
DWI: Diffusion-weighted imaging
ESS: Endometrial stromal sarcoma
Fat-Sat: Fat saturation
LMS: Leiomyosarcoma
MMMT: Carcinosarcoma/malignant mixed Mullerian tumor
MRI: Magnetic resonance imaging
STUMP: Smooth muscle tumors of uncertain malignant potential
T1WI: T1-weighted imaging
T2WI: T2-weighted imaging.
